# Cross-species transcriptional analysis reveals conserved and host-specific neoplastic processes in mammalian glioma

**DOI:** 10.1038/s41598-018-19451-6

**Published:** 2018-01-19

**Authors:** Nina P. Connolly, Amol C. Shetty, Jesse A. Stokum, Ina Hoeschele, Marni B. Siegel, C. Ryan Miller, Anthony J. Kim, Cheng-Ying Ho, Eduardo Davila, J. Marc Simard, Scott E. Devine, John H. Rossmeisl, Eric C. Holland, Jeffrey A. Winkles, Graeme F. Woodworth

**Affiliations:** 1 Department of Neurosurgery, University of Maryland School of Medicine, Baltimore, Maryland USA; 2 Institute for Genome Sciences, University of Maryland School of Medicine, Baltimore, Maryland USA; 3 Virginia Bioinformatics Institute and Department of Statistics, Virginia Tech, Blacksburg, Virginia USA; 4 Departments of Pathology and Laboratory Medicine, Neurology, and Pharmacology, Lineberger Comprehensive Cancer Center and Neuroscience Center, University of North Carolina, Chapel Hill, North Carolina USA; 5 Department of Pathology, University of Maryland School of Medicine, Baltimore, Maryland USA; 6 Marlene and Stewart Greenebaum Comprehensive Cancer Center, University of Maryland School of Medicine, Baltimore, Maryland USA; 7 Department of Microbiology and Immunology, University of Maryland School of Medicine, Baltimore, Maryland USA; 8 Department of Medicine, University of Maryland School of Medicine, Baltimore, Maryland USA; 9 Department of Small Animal Clinical Sciences, Virginia-Maryland College of Veterinary Medicine, Blacksburg, Virginia USA; 10 Wake Forest University Baptist Health Comprehensive Cancer Center, Brain Tumor Center of Excellence, Winston-Salem, North Carolina USA; 11 Fred Hutchinson Cancer Research Center, University of Washington, Seattle, Washington USA; 12 Department of Surgery, University of Maryland School of Medicine, Baltimore, Maryland USA; 13 Center for Vascular and Inflammatory Diseases, University of Maryland School of Medicine, Baltimore, Maryland USA

## Abstract

Glioma is a unique neoplastic disease that develops exclusively in the central nervous system (CNS) and rarely metastasizes to other tissues. This feature strongly implicates the tumor-host CNS microenvironment in gliomagenesis and tumor progression. We investigated the differences and similarities in glioma biology as conveyed by transcriptomic patterns across four mammalian hosts: rats, mice, dogs, and humans. Given the inherent intra-tumoral molecular heterogeneity of human glioma, we focused this study on tumors with upregulation of the platelet-derived growth factor signaling axis, a common and early alteration in human gliomagenesis. The results reveal core neoplastic alterations in mammalian glioma, as well as unique contributions of the tumor host to neoplastic processes. Notable differences were observed in gene expression patterns as well as related biological pathways and cell populations known to mediate key elements of glioma biology, including angiogenesis, immune evasion, and brain invasion. These data provide new insights regarding mammalian models of human glioma, and how these insights and models relate to our current understanding of the human disease.

## Introduction

Glioblastoma (GBM) remains a fatal disease with relatively few and marginally effective therapeutic options, despite years of concerted research effort and significant investment in new treatment strategies^1,2^. The current standard of care includes maximum safe surgical resection or biopsy followed by combined radiation and chemotherapy. This treatment protocol was established by Stupp and colleagues over 10 years ago, and median survival for GBM patients remains less than two years^3^.

A major obstacle to identifying new effective treatment strategies against GBM is accurately predicting which experimental therapies will counteract the disease in humans. In order to improve this predictive capability, a major research emphasis has been placed on developing representative experimental models of human glioma. These models have included patient-derived tumor cells and xenografts grown in immunocompromised hosts, mutagen-induced brain tumors and immortalized, transplantable cell lines, transgenic animal species and canines that develop spontaneous brain tumors, and genetically-engineered inducible brain tumors using viruses or other molecular modification systems^4–6^. Each of these experimental systems has specific advantages and disadvantages that depend in part on features of the model environment or tumor host^7^.

Human glioma is a complex ecosystem that develops exclusively within the central nervous system (CNS) and rarely metastasizes. This strongly implicates the host environment in the disease pathogenesis. Therefore, a critical element of accurate, predictive glioma models is the requirement for *in situ* tumor initiation and progression within the host CNS. While multiple experimental models exist that enable *in situ* tumor formation within the brain, little is known about the relative contributions of different host species to the disease initiation and patho- biological evolution. This information has important implications not only for representative modeling of human brain cancer, but also predictive testing of new treatment strategies. Autochthonous, genetically engineered, and spontaneous gliomas that exhibit *in situ* tumor formation in healthy and immunocompetent mammalian host species enable the study of this biological interplay throughout the formation and evolution of the disease. These tumors have been characterized in numerous mammalian species, including transgenic mice and rats, and dogs with naturally occurring glioma-canine gliomas having the distinct difference of not requiring experimental manipulation. The overlapping and species-specific contributions of the host background to the tumor biology remain relatively unexplored.

A particularly powerful and versatile modeling technology utilizes the replication competent avian-like sarcoma (RCAS) virus and its avian tumor virus A (TV-A) cell surface receptor inserted into the genome of the model host animal^8–13^. TV-A gene expression is controlled by a cell-type specific promoter (e.g. nestin), which is only activated in neural and glial progenitor cells – the cells implicated as brain tumor initiating cells (BTICs). Expression of the TV-A receptor on BTICs serves as a port of entry for TV-A-specific viruses (e.g. RCAS) engineered to carry genes linked to the development of GBM. Over-activation of oncogenes (e.g. platelet-derived growth factor-A (PDGF-A)) or functional loss of tumor suppressor genes (e.g. p53 or PTEN) induced using the RCAS/TV-A system leads to distinct tumor subtypes^9^. Experimental mouse models examining combinations of PDGF-A overexpression with p53 deficiency, or combined p53, NF1, and PTEN deficiency in nestin-positive BTICs, have shown histopathologic and genetic similarity to the human proneural and mesenchymal tumor subtypes, respectively^8–10,14^. Similar tumors initiated in transgenic TV-A rats display many of the key features found in human tumors, such as microvascular proliferation and brain invasion^15^. This suite of genetically engineered RCAS/TV-A models enables the study of inter-species and tumor subtype-specific biological differences.

In addition to these experimental models, domesticated dogs develop spontaneous gliomas with many of the key features of the human disease^6,16,17^. Dogs have cohabitated with humans for thousands of years, increasing the overlap of accumulated infectious and environmental exposures, which may contribute to spontaneous tumor formation in the brain and throughout the body. Dogs are also relatively outbred and live in less controlled, less sterile environments compared to laboratory animals. Increasingly, veterinary groups are partnering with clinical research teams to evaluate new therapies and compare findings in canines with those from human studies. Recently, the National Cancer Institute Comparative Brain Tumor Consortium has advocated for expanded study of tumor-bearing canine patients to further establish the overlap in human-canine oncogenic mechanisms and treatment responses, and to explore the possibility that this information may expedite the translation of new treatments into the clinic for both species^18^.

In this study, we set out to study the conserved and species-specific gene expression patterns and related signaling pathways present in rat, mouse, canine, and human gliomas. In order to accomplish this in the setting of the broad cellular and molecular heterogeneity present in human gliomas, we chose to focus this study on gliomas with a similar biological driver - up-regulation of the PDGF signaling axis. PDGF signaling in one of the strongest drivers of glioma initiation and evolution^9,19^ and the PDGF receptor-alpha (PDGFRA) tyrosine kinase is frequently overexpressed in human gliomas^20,21^. The working hypothesis for the study was that similar core neoplastic alterations are present in each mammalian species; however, subtle differences in tumor host anatomy and physiology likely lead to unique biological contributions related to the tumor host species.

## Methods

### Rodent brain tumor samples, RNA isolation, and gene expression analysis

All experiments were conducted in accordance with protocols approved by the University of Maryland School of Medicine (rat) and University of Washington School of Medicine (mouse) Institutional Animal Care and Use Committees (IACUC) and followed NIH guidelines for animal welfare. Gene expression data were derived from Ntv-a mouse and rat tumors initiated by the same RCAS-mediated oncogenic transformations: PDGF-A overexpression and p53 depletion^9,15^. Tumors from the mouse (n = 4) and rat (n = 4) were harvested at the time of euthanasia due to disease progression and immediately processed. Normal control rat brain tissue (n = 4) was collected from age- and sex-matched animals in the same hemisphere as the tumors. Control mouse brain tissue (n = 4) was collected from the frontal lobe. Gene expression analysis of murine brain and brain tumor tissue was performed using the Illumina BeadChip platform as described^9^. Rat tissue was analyzed for gene expression changes by DNA microarray analysis in collaboration with the Biopolymers-Genomics Core Facility at the University of Maryland School of Medicine. Briefly, RNA was isolated using RNeasy Mini Kit (Qiagen, Hilden, Germany). Samples were hybridized overnight in GeneChip Hybridization Oven 640 using the Rat Transcriptome Array 1.0 kit (Affymetrix, Santa Clara, California). After hybridization, the samples were washed and stained using GeneChip Fluidics Station 450 (Affymetrix). CEL files were generated after scanning with the Affymetrix GeneChip Scanner 7 G.

### Canine brain tumor samples, RNA isolation, and gene expression analysis

All experiments were conducted in accordance with protocols approved by the been reviewed and approved by the Virginia Tech IACUC and the board of the Veterinary Teaching Hospital and followed NIH guidelines for animal welfare. Brain tumor samples from dogs (n = 5) with naturally occurring prosencephalic astrocytic gliomas were analyzed. Owner’s provided written consent for their dog’s tissues to be harvested and banked in a biospecimen repository and used for research purposes (IACUC 13-153-CVM). Brain tumor samples were obtained antemortem using excisional or CT-guided stereotactic needle biopsy techniques in 3 dogs or at the time of necropsy in 2 dogs. Immediately upon harvesting, tissue samples were frozen in liquid nitrogen and stored at −80 °C until analysis. Control brain tissues were obtained from purpose-bred beagles (n = 5) with no clinical, MRI, or histologic evidence of brain disease and euthanized for reasons unrelated to this study. Control dogs were maintained under a separate approved protocol (IACUC 07-060-CVM). Control brain tissue samples were dissociated using a dissecting microscope and pooled to form grey matter samples originating from the parietal, temporal, and frontal cerebral cortices and white matter samples from the internal capsule, centrum semiovale, and corpus callosum. For the purposes of this study, H&E, Olig-2, and GFAP stained slides from each canine tumor were reviewed by a single pathologist blinded to the original diagnoses who then typed and graded each tumor according to WHO criteria. Tumor types and grades included Grade II/III astrocytoma (n = 2), Grade IV astrocytoma (GBM; n = 2), and Grade III oligoastrocytoma (n = 1). The canine patients included Boston terrier, male-neutered (MN), 6 years old; American Bulldog, female-spayed (FS), 8 years old; Boxer, MN, 7 years old; Boxer, MN, 5 years old; Labrador mix, FS, 8 years old. RNA was isolated from tumor cells using the RNAeasy Mini Kit and QIAshredder (QIAGEN, Valencia, CA, USA), according to the manufacturer’s protocol. The RNA concentration was determined using a NanoDrop ND-1000 UV-Vis spectrophotometer (NanoDrop Technologies, Wilmington, DE) and quality was measured using a 2100 Bioanalyzer (Agilent, Santa Clara, CA, USA) with the 6000 chip.

Microarray gene expression profiling was performed at the Core Laboratory Facility of the Virginia Bioinformatics Institute. Approximately 4 µg of RNA was labeled using the Affymetrix labeling protocol (Affymetrix). The protocol described in GeneChip Eukaryotic Small Sample Target Labeling Assay Version II (www.affymetrix.com) was used to generate, amplify, and label biotinylated cRNA. The cRNA samples were then hybridized to Affymetrix GeneChip Canine Genome 2.0 microarrays (Affymetrix) following the manufacturer’s protocol. Raw data were obtained with the high-density GeneChip Scanner 3000. Quality control was performed using Affymetrix’s recommended measures and evaluations enabled by the software packages made4, affy and affyPLM. All chips passed quality control. Of the 43,035 probesets on this chip version, 33,477 remained after removal of control probes and probes called absent (MAS5) on all chips.

### Comparative differential gene expression analysis – rat, mouse, canine

The rat Affymetrix microarray samples were analyzed using the R ‘oligo’ and ‘limma’ analytical packages to determine the differentially expressed genes between 4 PDGFR-A-overexpressing tumors and 4 normal brain samples. The microarray intensity values were normalized using the ‘robust multichip average’ (rma) normalization method implemented in oligo. Outliers were detected using principal component analysis (PCA) and excluded from downstream analysis. Differentially expressed genes between tumor and normal conditions were assessed using linear models and empirical Bayes procedures provided within limma. The mouse Illumina microarray samples were analyzed using the R ‘lumi’ and ‘limma’ analytical packages to determine the differentially expressed genes between 4 tumors and 4 normal brain samples. The microarray intensity values were normalized using methods implemented in lumi. Outliers were detected using PCA and excluded from downstream analysis. Differentially expressed genes between tumor and normal samples were assessed using linear models and empirical Bayes procedures provided within limma. The canine Affymetrix microarray samples were analyzed using the R ‘oligo’ and ‘limma’ analytical packages to determine differentially expressed genes between 5 tumors, 5 normal white matter and 5 normal grey matter samples. Gray and white matter control samples and 3 PDGFRA-overexpressing tumors were utilized in our analysis. The microarray intensity values were normalized as described above for the rat tumor analysis. The per-gene p-values were adjusted for multiple testing using the Benjamini-Hochberg procedure, which controls for false discovery rate (FDR). The significant differentially expressed genes for all three datasets were detected using an adjusted p-value cut-off less than 5% and a minimum of 1.5X fold-change cut-off and further illustrated as heatmaps created using the R package ‘gplots’. The heatmaps show the gene expression values for each of the significantly differentially expressed genes across each sample in the dataset and the hierarchical clustering of samples is illustrated as a column dendrogram.

### Human brain tumor dataset and analysis

We downloaded gene expression read count values for 400 normal brain samples from GTEx Consortium Data Portal (GTEx_Analysis_v6p_RNA-seq_RNA-SeQCv1.1.8) comprised of 114 anterior cingulate gyrus tissue samples, 148 cortex tissues samples and 138 frontal cortex tissue samples, comprising mixed gray and white matter. For human GBM samples, we downloaded gene expression read count values for 155 primary GBM tumors analyzed with RNA sequencing available on the The Cancer Genome Atlas (TCGA) Data Portal (https://gdc.cancer.gov/). The gene expression read count values for each sample were normalized for sample library size and gene expression dispersion using methods developed in the R analytical package ‘DESeq’, commonly used to analyze gene expression datasets generated from RNA-sequencing techniques. In order to ensure that the human dataset was comparable to the PDGFR-α overexpressing rat, mouse and canine glioma datasets, we only retained 109 GTEx normal samples with low PDGFRA expression and 65 TCGA tumors with PDGFRA expression greater than twice the maximum expression of PDGFR-α in the GTEx normal samples. While tumor metabolism and IDH mutations are key considerations in gliomas, for this study no IDH mutated human gliomas were included as the canine glioma IDH status was not available and the rodent tumors were IDH wildtype. The final set of samples were utilized to compare between tumor and normal samples using a negative binomial general linear model as implemented in the DESeq R package. The per-gene p-values were adjusted for multiple testing using the Benjamini-Hochberg procedure, which controls for false discovery rate (FDR). The significant differentially expressed genes were detected using an adjusted p-value cut-off less than 5% and a minimum 2X fold-change cut-off and further illustrated as heatmaps created using the R package ‘gplots’. The heatmaps show the gene expression values for each of the significantly differentially expressed genes across each sample in the dataset and the hierarchical clustering of samples is illustrated as a column dendrogram.

### Comparative differential gene expression analysis of rat, mouse, canine datasets to human datasets

Using the annotation files available for each of the glioma microarrays and the Eensemble gene annotation for the human glioma dataset, we compared each set of significant differentially expressed genes (DEGs) detected from the analysis of the microarray datasets to those detected from the analysis of the human glioma dataset. DEGs were categorized into 4 bins, namely, (i) genes up-regulated in both the microarray dataset and the human glioma dataset, (ii) genes down-regulated in both the microarray dataset and the human glioma dataset, (iii) genes up-regulated in the microarray dataset and down-regulated in the human glioma dataset, and (iv) genes down-regulated in the microarray dataset and up-regulated in the human glioma dataset. These DEGs were illustrated as scatter-plots using the R package ‘ggplot2’ showing the log_2_ (fold-change) values for each DEG from the microarray and human glioma datasets on individual axes. The proportion of DEGs in each bin were computed as the number of DEGs in the bin compared the total number of DEGs in all four bins. A Venn diagram, generated using the R package ‘gplots’ was used to display the number of increased and decreased DEGs in the four mammalian tumor types.

### Pathway enrichment of GBM signature genes

To examine the relationship between the overlapping and species-specific DEG patterns, we selected a subset of 95 up-regulated genes and 30 down-regulated genes from the lists of overlapping up- and down-regulated genes, known to play a role in glioma biology based on an extensive literature search (See Supplementary Data for gene list and PubMed IDs). The DEG data for these 125 glioma-associated genes from all four species were then utilized to determine enrichment of functional pathways using the Database for Annotation, Visualization and Integrated Discovery (DAVID) web-based tool. Enriched pathways determined from DAVID analysis were further filtered using an FDR cut-off ≤5% and clustered based on DAVID annotation into 49 clusters with enrichment scores ranging from 2 to 12. These clusters were further categorized into 7 enriched pathway groups, namely, (i) angiogenesis, (ii) cell cycle and apoptosis, (iii) DNA repair, (iv) immune signaling, (v) migration and invasion, (vi) signal transduction, and (vii) stem cell and development. The maximum enrichment score associated with each pathway group was illustrated as horizontal barplot using the R package ‘ggplot2’. We determined the genes associated with each of the 7 enriched super pathway groups based on the results from the DAVID pathway cluster analysis and selected 66 representative genes (7 associated with angiogenesis, 5 associated with cell cycle and apoptosis, 4 associated with DNA repair, 9 associated with immune signaling, 15 associated with migration and invasion, 18 associated with signal transduction, and 8 associated with stem cell and development). The log_2_ (fold-change) values for these 66 genes across all 4 species were determined and illustrated as horizontal barplots using the R package ‘ggplot2’.

### Validation series using immunofluorescence microscopy

Rodents were euthanized with induction of general anesthesia followed by exsanguination using transcardiac perfusion of cold phosphate buffered saline (PBS). The brain was rapidly extracted and sectioned into 2-mm thick sections. Tumor-containing sections were fixed in 4% formalin for 24 h and transferred to 70% ethanol for 24 hours before embedding in paraffin. Tumors for human patients, following informed consent, were collected in accordance with University of Maryland Institutional Review Board (HP-00066139) and were analyzed in a de-identified manner. Human tumor tissue included primary GBM samples collected at surgery and canine tumor samples that were collected at surgery or necropsy. Samples were fixed in 10% neutral buffered formalin before embedding in paraffin. Fixed tissues were mounted in paraffin blocks using the Leica EG 1160 embedding center (Leica Microsystems, Wetzlar, Germany) and then sectioned in 5 µm slices oriented in the coronal plane. Sections were stained and examined using standard histopathological techniques (hematoxylin & eosin, PDGFR-α immunohistochemistry (1:200; sc-338, Santa Cruz Biotechnology, Dallas, TX) and reviewed with a neuropathologist (C.H.). Antigen retrieval was performed using either Bond Epitope Retrieval Solution 1 (pH ~6) or Bond Epitope Retrieval Solution 2 (pH ~9) (Leica Microsystems) at 99–100 °C for 20–30 min.

For immunofluorescence, sections from paraffin embedded normal brain and tumor tissues were obtained. Canine spleen, rat kidney, and mouse skin tissues were also processed in a similar fashion. Sections were de-paraffinized in xylene 30 min, 100% ethanol 15 min, 95% ethanol 15 min, 70% ethanol 15 min, ultrapure water 5 min, and PBS 5 min. Microwave antigen retrieval was performed in IHC-TEK epitope retrieval solution (IW-1100; IHC World LLC, Ellicott City, MD). Sections were microwaved for 2 min, followed by 3 min rest; this was repeated a total of 3 times. Tissues were then blocked for 1 h in 2% donkey serum with 0.2% Triton X-100 and labeled overnight at 4 °C with primary antibodies against CD44 (rabbit polyclonal, 1:200; ab157107, Abcam, Cambridge, MA), DNA Topoisomerase 2-α (rabbit monoclonal, 1:200; ab52934, Abcam), TNFRSF12A (rabbit polyclonal, 1:200; bs-2493R, Bioss, Woburn, MA), BMP-7 (rabbit polyclonal, 1:200; ab27569, Abcam), or Periostin (rabbit polyclonal, 1:200; ab83739, Abcam). Alexa Fluor 500- or fluorescein isothiocyanate-conjugated secondary antibodies (donkey polyclonal) were applied, and tissues were coverslipped with ProLong Gold antifade reagent (P36930; Thermo Fisher Scientific Inc., Waltham, MA). Specific labeling was confirmed by omission of primary antibody. Immuno-labeled tissues were visualized with epifluorescence microscopy (Nikon Eclipse 90i; Nikon Instruments Inc., Melville, NY).

### Gene set enrichment analysis

Gene set enrichment analysis (GSEA)^22^ was implemented using the GSEA software developed by the Broad Institute. The differentially expressed genes identified for each of the four GBM datasets were ranked based on the log2 (fold-change) and the adjusted P-values such that the up-regulated genes would be at the top of the list while the down-regulated genes would be at the bottom of the list. Each of the pre-ranked gene lists were compared to 14 gene signature sets downloaded from the Engler *et al*.^23^, representing multiple cell subtype-specific signatures and immune response-related gene sets, and three gene signature sets downloaded from Cahoy *et al*.^24^ representing gene sets enriched in astrocytes, neurons and oligodendrocytes. For all GSEA analysis, 1000 permutations were performed to generate significance p-values that were further corrected for multiple hypothesis testing to generate false discovery rate (FDR) values for each gene set. A FDR value of less than 25% was considered to be statistically significant as suggested by the developers. The normalized enrichment values reported by GSEA were utilized to determine enrichment with up- or down-regulated genes.

## Results

We found that transgenic TV-A mice and rats co-infected with PDGF-A and p53 shRNA RCAS viruses develop intracranial tumors that overexpress PDGFRA (Supplementary Figure 1). Therefore, in order to make meaningful comparisons between intrinsically heterogeneous tumors, we concentrated our cross-species glioma gene expression analysis to only those tumors with high levels of PDGFRA gene expression. Accordingly, we first screened the five canine spontaneous glioma samples for overexpression of this gene and found three that met this selection criteria (Supplementary Figure 1).

**Figure 1:**
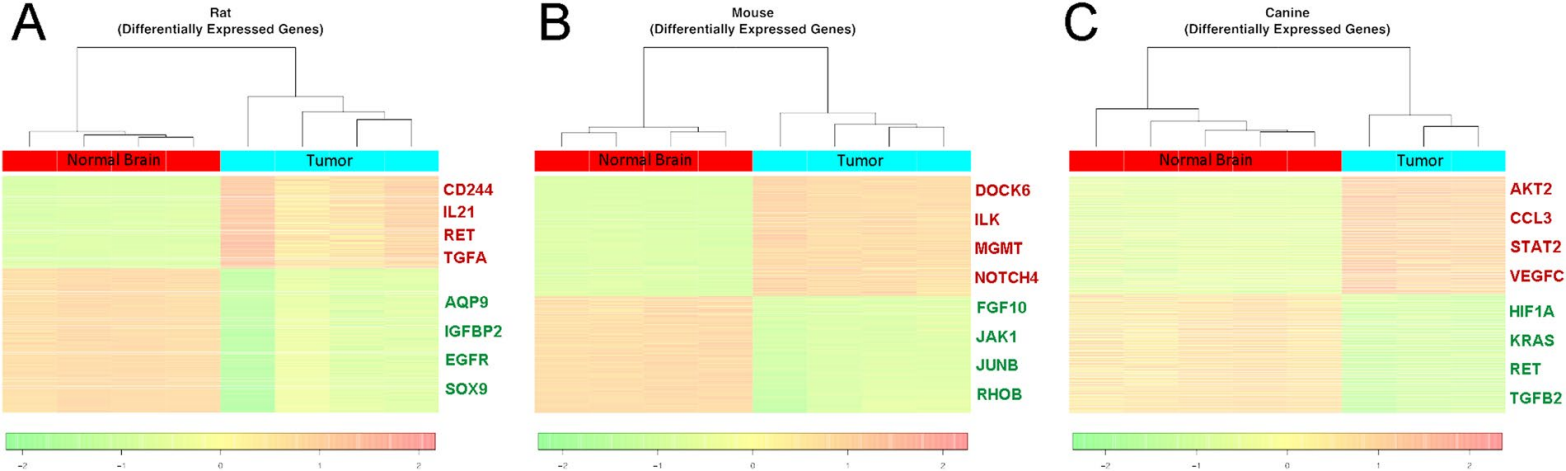
Differential gene expression in PDGFRA-overexpressing rat, mouse, and canine *in situ*-formed gliomas. Heatmaps representing the degree of variation and clustering of gene expression between control, normal brain tissue (red bar) and tumor tissue (blue bar). (**A**) RCAS/TV-A rat gliomas: there were 1,710 DEGs in total; this included 661 up-regulated genes and 1,049 down-regulated genes. Several examples of glioma-associated DEGs that were upregulated or downregulated specifically in the rat model are listed. (**B**) RCAS/TV-A mouse gliomas: there were 3,223 DEGs in total with 1,658 up-regulated and 1,565 down-regulated in tumor tissue compared to normal brain. Several examples of glioma-associated DEGs that were upregulated or downregulated specifically in the mouse model are listed. (**C**) Spontaneous canine glioma: there were 4,166 DEGs in total with 2,109 up-regulated and 2,057 down-regulated in tumor tissue compared to normal brain. Several examples of glioma-associated DEGs that were upregulated or downregulated specifically in dogs are listed. [Scale bar representing graded fold change in gene expression: red = increased expression, green = decreased expression].

Rat, mouse, and canine normal brain and brain tumor RNA samples were isolated and gene expression analysis was performed. The heatmaps show the gene expression values for each of the significantly DEGs across each sample in the dataset and the hierarchical clustering of samples is illustrated as a column dendrogram (Fig. 1). The rat model had 1,710 DEGs in total; this included 661 up-regulated genes and 1,049 down-regulated in tumor tissue compared to normal brain (Supplementary Table 1). Glioma-associated DEGs CD244, IL21, RET, TGFA were up-regulated and AQP9, IGFBP2, EGFR, SOX9 were down-regulated specifically in the rat (Fig. 1A). The mouse model had 3,223 DEGs in total with 1,658 up-regulated and 1,565 down-regulated in tumor tissue compared to normal brain (Supplementary Table 2). Glioma-associated DEGs DOCK6, ILK, MGMT, NOTCH4 were up-regulated and FGF10, JAK1, JUNB, RHOB were down-regulated uniquely in the mouse (Fig. 1B). The canine model had 4,166 DEGs in total with 2,109 up-regulated and 2,057 down-regulated in tumor tissue compared to normal brain (Supplementary Table 3). Glioma-associated DEGs AKT2, CCL3, STAT2, VEGFC were up-regulated and HIF1A, KRAS, RET, TGFB2 were down-regulated specifically in the dogs (Fig. 1C).

**Figure 2:**
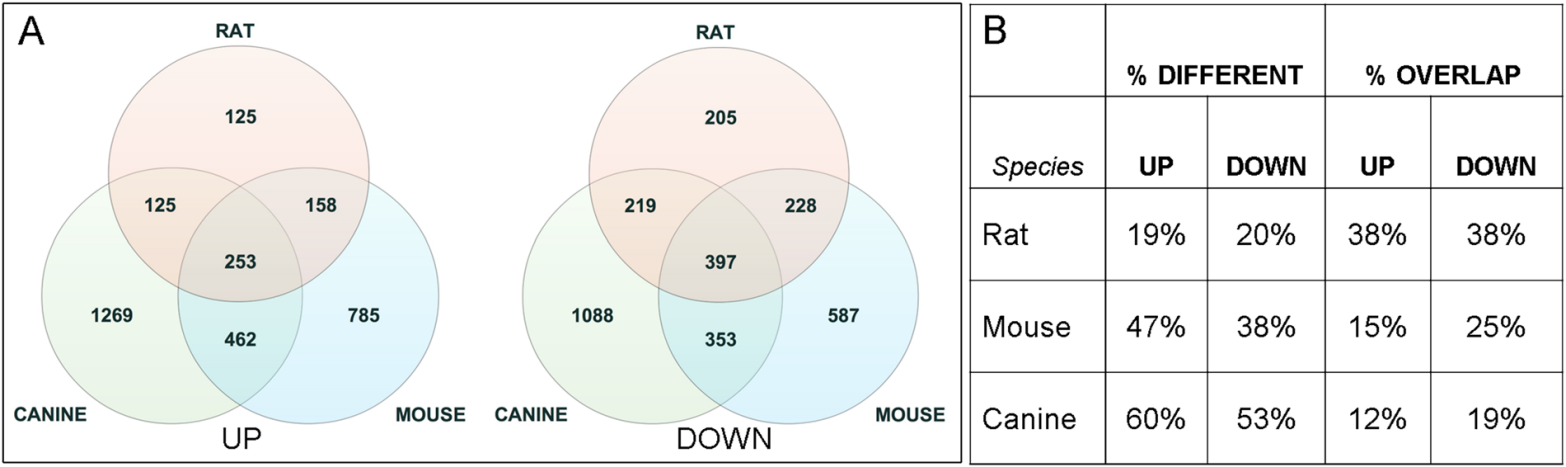
Rat, mouse, and canine glioma differentially expressed genes (DEG) Venn diagrams and data summary table. (**A**) Relative overlap of increased (UP) and decreased (DOWN) DEGs in rat, mouse, and canine PDGFRA-overexpressing glioma. (**B**) The unique and overlapping DEGs were compared by calculating the percentage of unique DEGs in the total DEG set for that species (% different) and the percentage of the common glioma DEGs represented within the total DEG set for that species (% overlap). The rat tumors had the lowest percentage of different DEGs and the greatest percent of common glioma genes represented within the total rat DEG set. The canine tumors showed the highest percent difference and the lowest percent overlap.

The rat, mouse and canine DEG sets were compared with one another to determine the extent of overlap between the increased and decreased genes for the three species. The results are illustrated in Venn diagrams (Fig. 2A). There were 253 overlapping genes with elevated expression, including CD44 and STAT3, and 397 overlapping genes with decreased expression, including OPALIN and MAPK9. The unique and overlapping DEGs were compared by calculating the percentage of unique DEGs in the total DEG set for that species (% different) and the percentage of the common glioma DEGs represented within the total DEG set for that species (% overlap) (Fig. 2B). The rat tumors had the lowest percentage of different DEGs and the greatest percent of common glioma genes represented within the total rat DEG set. The canine tumors showed the highest percent difference and the lowest percent overlap.

**Figure 3:**
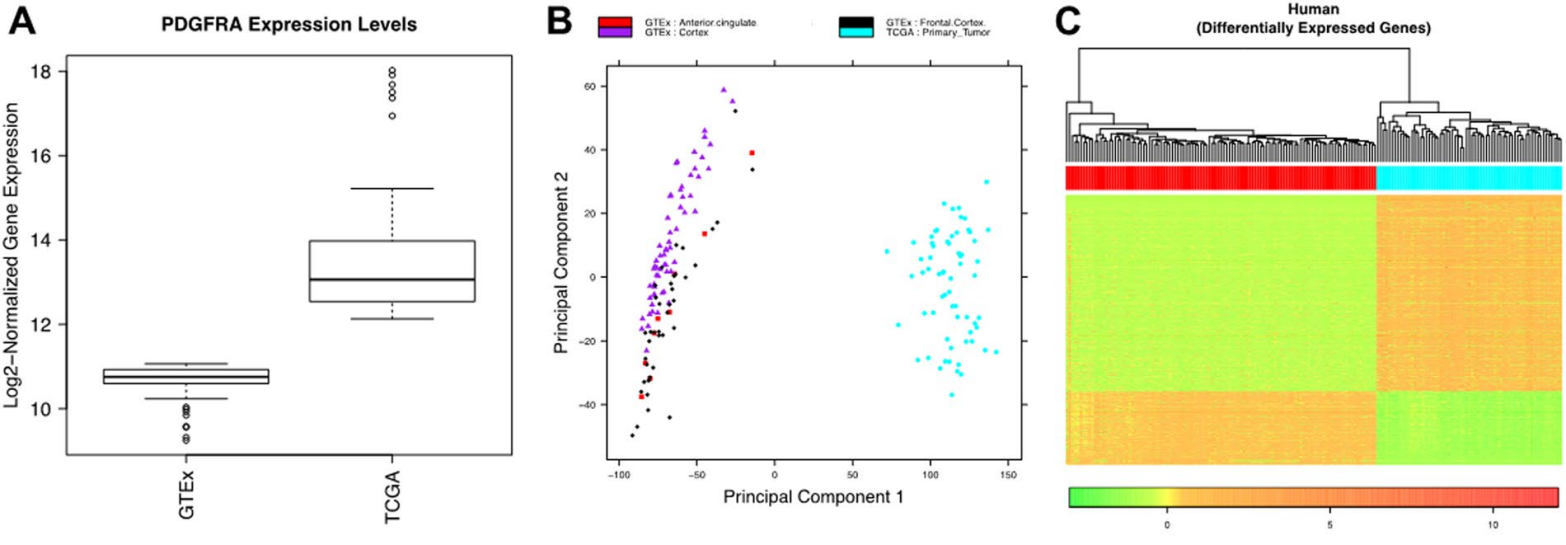
Differential gene expression in PDGFRA-overexpressing human glioma. (**A**) PDGFRA expression levels in normal brain and tumor samples derived from publicly available GTEx and TCGA gene expression data plotted as box and whisker plots. Whiskers of the plot represent the 1.5 IQR of the upper quartile and 1.5 IQR of the lower quartile score (Tukey boxplot) for each expression cluster. Bar in box represents the median value for each group. Top and bottoms of the box represent the 25^th^ and 75^th^ percentile, respectively, of the values for each group. (**B**) Principal component analysis demonstrating clustering of normal brain specimens including anterior cingulate tissue (red squares), cerebral cortex tissue (purple triangles), frontal cortex tissue (black diamonds), and brain tumor (blue circles) samples. (**C**) Heatmap representing the degree of variation in gene expression between control, normal human brain tissue (red bar) and human tumor tissue (blue bar). [scale bar: red = increased expression, green = decreased expression].

For the human glioma analysis, we used 109 normal brain (anterior cingulate gyrus and frontal cortex tissue) samples from the Genotype Tissue Expression (GTEx) Consortium Data Portal (www.gtexportal.org) to estimate baseline PDGFRA values. The human glioma gene expression data from 155 primary GBM tumors in The Cancer Genome Atlas (TCGA) Data Portal was analyzed and 65 tumors were selected for the DEG comparison based on elevated (≥10 log2a change) PDGFRA gene expression (Fig. 3A). Principal component analysis showed distinct clustering of the normal brain samples of various types separate from the tumor tissue samples (Fig. 3B). DEG heatmaps further characterized this sample clustering and revealed 6,888 DEGs in total with 3,888 up-regulated and 3,000 down-regulated in tumor tissue compared to normal brain (Fig. 3C, Supplementary Table 4).

**Figure 4:**
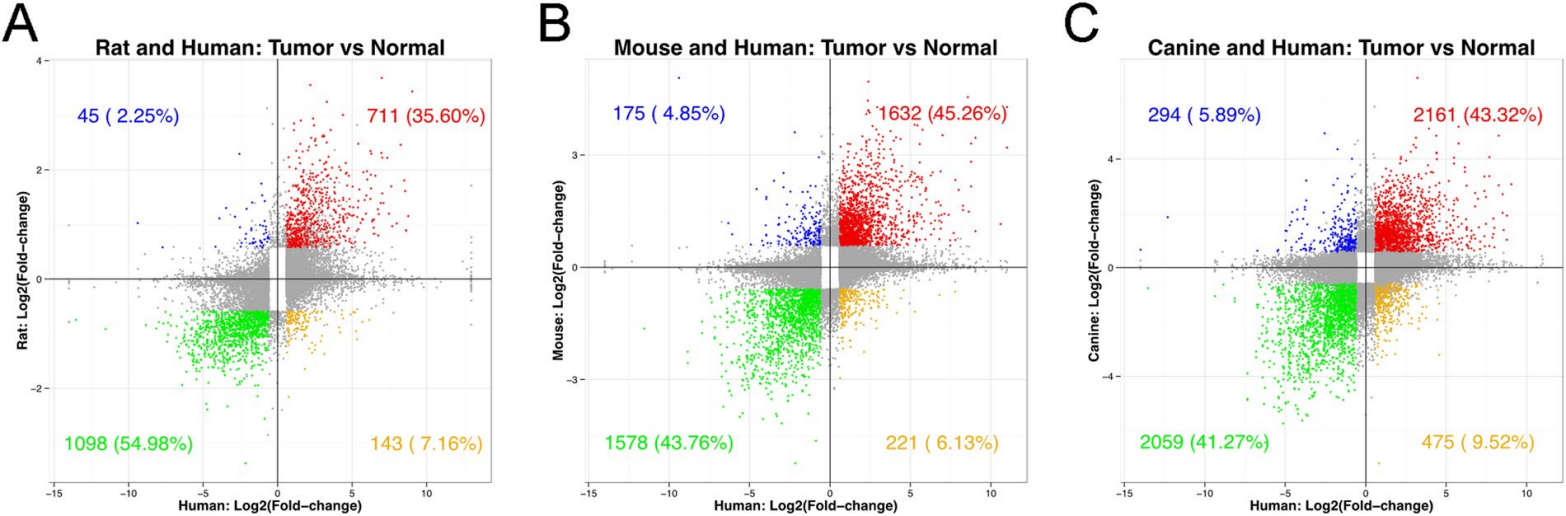
Non-human – human glioma DEG comparisons. (**A**) Rat-Human DEG comparison (**B**) Mouse-Human DEG comparison (**C**) Canine-Human DEG comparison. The four quadrants represent increased expression species X, decreased expression human (blue dots); increased expression species X, increased human (red dots); decreased expression species X, decreased expression human (green dots); decreased expression species X, increased expression human (yellow dots), where X is either rat, mouse, or canine. Gray dots represent DEGs that did not reach statistical significance in either human or species X. In each comparative case, the total directionally-overlapping, significant DEGs was found to be greater than 80% (red plus green dots).

The human PDGFRA overexpressing glioma DEG dataset was then compared to the rodent and canine PDGFRA overexpressing glioma DEG datasets separately. The DEGs were categorized into 4 bins: increased expression species X, decreased expression human (blue dots); increased expression species X, increased human (red dots); decreased expression species X, decreased expression human (green dots); decreased expression species X, increased expression human (yellow dots), where X is either rat (Fig. 4A), mouse (Fig. 4B), or canine (Fig. 4C). Of all the significant DEGs shared by rats and human, 35.6% are increased and 54.9% are decreased, as compared to mouse and humans in which 45.3% were increased and 43.8% were decreased, and canine and humans where 43.3% were increased and 41.3% were decreased (Supplementary Table 5). In each comparative case, the total directionally-overlapping, significant DEGs was found to be greater than 80% (red plus green dots). Genes not reaching significance in either human or species X are depicted in gray on each graph and were not included in the analysis.

**Figure 5:**
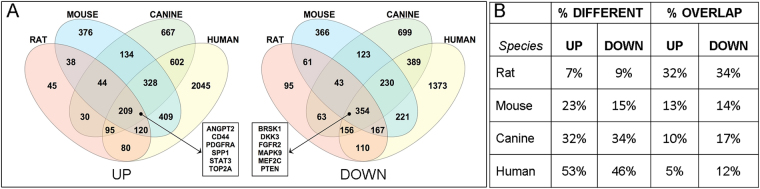
Four-species DEG Venn diagrams and data summary table. (**A**) Relative overlap of increased (UP) and decreased (DOWN) differentially expressed genes in rat, mouse, canine and human PDGFRA-overexpressing glioma. Six examples of commonly up-regulated or down-regulated DEGs are listed in the boxes. (**B**) The unique and overlapping DEGs were compared by calculating the percentage of unique DEGs in the total DEG set for that species (% different) and the percentage of the common glioma DEGs represented within the total DEG set for that species (% overlap). The rat tumors showed the smallest percentage of unique DEGs, and the largest percent overlap of the common DEGs within the total rat DEG set. The human and canine tumors showed the greatest percentage of unique DEGs and lowest percent overlap of the common DEGs.

The human PDGFRA overexpressing glioma DEG dataset was then compared to the rodent and canine PDGFRA overexpressing glioma DEG datasets together. Venn diagrams show the number of unique and overlapping increased and decreased DEGs for each species (Fig. 5A). A complete list of DEGs in each group is provided as Supplementary Data. The four species share 209 increased and 354 decreased DEGs. Several examples of DEGs that contribute to various aspects of gliomagenesis include the up-regulated genes encoding PDGFRA (as expected), ANGPT2, CD44, SPP1, STAT3, and TOP2A, and the down-regulated genes encoding BRSK1, DKK3, FGFR2, MAPK9, MEF2C, and PTEN. Similar to the non-human species only comparison, the rat tumors showed the smallest percentage of unique DEGs, and the largest percent overlap of the common DEGs within the total rat DEG set. The human and canine tumors showed the greatest percentage of unique DEGs and lowest percent overlap of the common DEGs (Fig. 5B).

**Figure 6:**
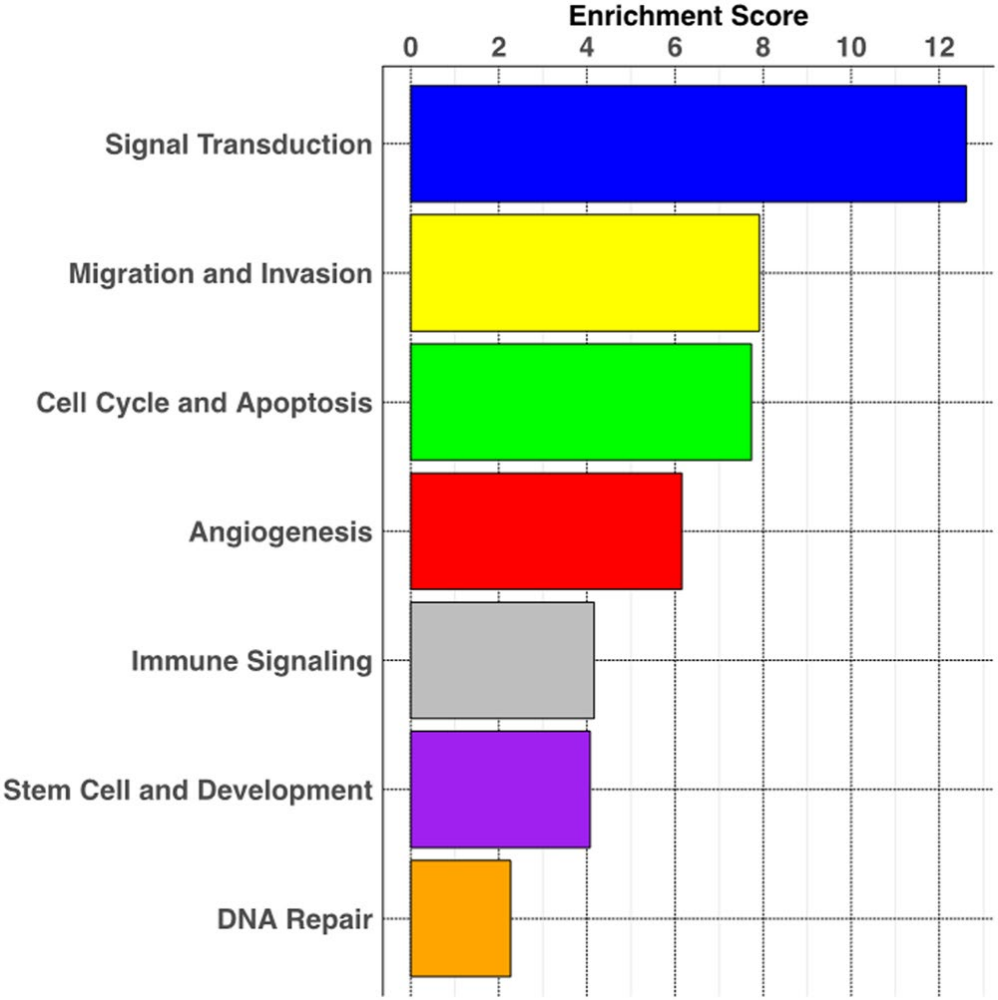
Pathway enrichment analysis. The DEG data for the 125 glioma-associated gene set for all the species was inputted into the DAVID function annotation algorithm. The enriched DEG sets were categorized into seven enriched pathway groups, and enrichment score was calculated for each pathway group.

**Figure 7:**
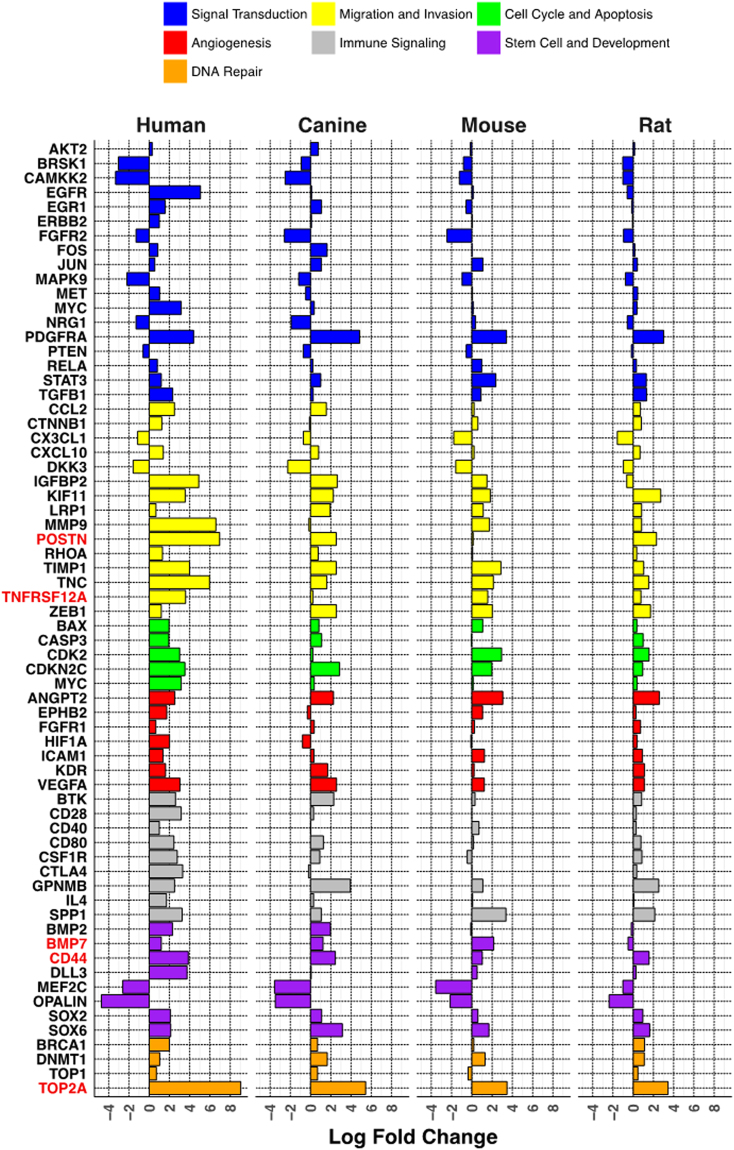
Comparative analysis of selected glioma signature gene expression patterns. A representative subset of the glioma-associated gene set used for the analysis in Fig. 6 was plotted by their log-fold change in each species. The individual genes were grouped by the predominant pathway to which they contributed. The expression pattern of the genes highlighted in red was analyzed at the protein level by immunofluorescence (Supplementary Figure 2).

To determine the potential functional implications of the observed DEG patterns, genes previously shown to play an important role in glioma biology were selected from the DEG datasets. One hundred twenty-five glioma-associated genes were entered into the DAVID function annotation algorithm (Supplementary Table 6). The enriched pathways determined from DAVID analysis were categorized into seven groups, including angiogenesis, cell cycle and apoptosis, DNA repair, immune signaling, migration and invasion, signal transduction, and stem cell and development (Fig. 6). An enrichment score was determined for each pathway group, which showed the greatest alterations in the signal transduction and migration/invasion pathways, and the least alteration in the DNA repair pathways. The 125 glioma-associated genes were next distributed between these pathways based on the DAVID pathway cluster analysis (Supplementary Table 7). Sixty-six glioma-associated genes, listed along the y-axis, are shown with their respective log-fold changes (x-axis) across all 4 species to show the variation within specific functional pathways (Fig. 7). Some genes, for example, CD44, TOP2A, and ANGPT2, show similar overexpression across species; however, multiple genes are marked by variable degrees of overexpression and contrasting directionality. Some specific notable gene expression differences included: epidermal growth factor receptor (EGFR) was only highly expressed in the human PDGFRA-overexpressing tumors; early growth response 1 (EGR1) expression was only increased in human and canine tumors, hypoxia-inducible factor 1-α (HIF1A), delta-like canonical notch ligand 3 (DLL3), cytotoxic T-lymphocyte associated protein 4 (CTLA4), and tumor necrosis factor receptor superfamily 12 A (TNFRSF12 A), also known as fibroblast growth factor inducible 14 (Fn14), were not differentially expressed in canine gliomas. Mice did not show differential expression of periostin (POSTN), colony stimulating factor receptor 1 (CSFR1) or topoisomerase 1 (TOP1); and rats were lacking differential expression of insulin growth factor binding protein 2 (IGFBP2) and bone morphogenetic protein 7 (BMP-7).

In order to evaluate whether the observed glioma transcriptional patterns translated to protein expression patterns, several genes with shared or variable expression across the four species were selected (Fig. 7; see red highlighted genes) for protein analysis using immunofluorescence (IF) microscopy. All four tumor types showed significantly increased expression of the CD44 transcript, which encodes a marker for reactive and stem-like glioma cells^25^ and is frequently overexpressed in high-grade glioma^26^. IF analysis confirmed the presence of the CD44 protein in all four tumor types with minimal staining in normal brain tissue (Supplementary Figure 2A). Similarly, the DNA topoisomerase 2A (TOP2A) gene, which encodes an enzyme most frequently involved in cell cycle progression^27^ and is often overexpressed in proliferating glioma cells and GBM tumors^28–30^, was over-expressed in all four species. IF analysis using TOP2A antibody also showed strong expression of TOP2A in the four tumor types (Supplementary Figure 2B).

Other DEGs showed variable expression across the four species. In order to validate these transcriptional observations, the expression pattern of several of the corresponding protein products was assessed by IF microscopy. The DEGs examined included (1) TNFRSF12A (Fn14), which encodes a cell surface protein that binds the ligand TWEAK and is over-expressed in many human solid tumor types including GBM^31,32^; (2) BMP-7, which encodes a member of the TGF-β superfamily implicated in glioma cell growth control and stem-like glioma cell properties^33–35^; and (3) POSTN, which encodes a secreted matricellular protein overexpressed in GBM that binds integrins and plays a role in macrophage recruitment and glioma cell invasion^36–38^. In agreement with the mRNA expression analysis data, TNFRSF12A expression was not detected in canine tumor tissue (Supplementary Figure 2C), BMP-7 expression was not detected in rat tumor tissue (Supplementary Figure 2D), and POSTN expression was not detected in mouse tumor tissue (Supplementary Figure 2E). Positive control tissues were used to validate that the TNFRSF12A, BMP-7 and POSTN antibodies used in this analysis were able to detect the canine, rat and mouse proteins, respectively (Supplementary Figure 3).

**Figure 8:**
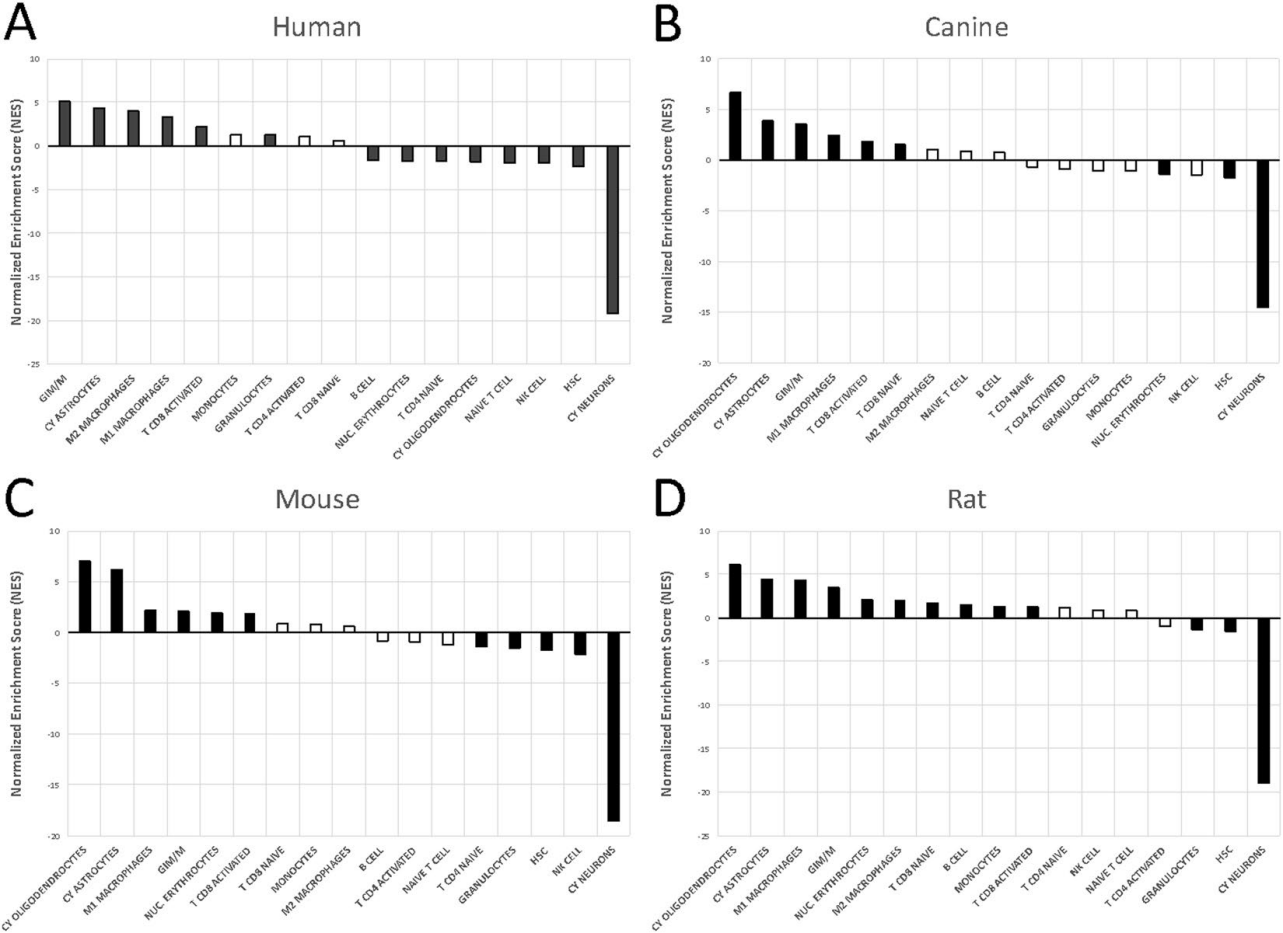
Gene set enrichment analysis. The DEG datasets were compared using GSEA incorporating cell type signatures for brain and infiltrating cell populations. (**A**) The human tumors showed an astrocytic predominance, whereas the three non-human species, (**B**) Canine, (**C**) Mouse, and (**D**) Rat, showed a mixed contribution of astrocytic and oligodendrocytic elements. Consistent with the expansion of glial components and displacement of neuronal components in gliomas, there was a significant decrease in the enrichment of the neuronal cell signature in tumors from all four species. Also, each species demonstrated evidence of activated cytotoxic T-lymphocytes (CD8+) and glioma-infiltrating macrophages and microglia (GIM/Ms). Only the human and rat tumors showed enrichment of the M2 macrophage signature. Black bars = FDR value < 25%; White bars = FDR ≥ 25%. [GIM/M: glioma-infiltrating macrophages and microglia; CY: Cahoy signatures; HSC: hematopoietic stem cell].

To further explore the implications of the observed transcriptional patterns, the DEG datasets were applied in a gene set enrichment analysis (GSEA)^22^ utilizing cell type signatures to compare the relative contributions of various tumor-host cell populations across the four species. The cell type signatures included those related to intrinsic CNS populations and those known to infiltrate into the brain in the setting of neoplastic and inflammatory conditions. The three non-human species showed a mixed contribution of astrocytic and oligodendrocytic elements compared to the human tumors (Fig. 8B–D), which showed more astrocytic predominance (Fig. 8A). This finding supports the concept that PDGFRA-overexpressing gliomas derive at least in part from the oligodendroglial lineage. It is possible that the human tumor samples and gene expression data contained a broader and more heterogeneous mix of glial tumors, diluting the oligodendroglial contribution in the human analysis. Consistent with the expansion of glial components and displacement of neuronal components in gliomas, there was a significant decrease in the enrichment of the neuronal cell signature in tumors from all four species. Each species demonstrated evidence of activated cytotoxic T-lymphocytes (CD8+) and glioma-infiltrating macrophages and microglia (GIMs), confirming these notable findings in human GBM^39^. Interestingly, only the human and rat tumors showed enrichment of the M2 macrophage signature, suggesting important variability in the GIM populations across the four species (Fig. 8A,D).

## Discussion

In this study, we investigated the differences and similarities in glioma characteristics based on differential gene expression patterns present in *in situ*-formed tumors derived from four mammalian species: rats, mice, dogs, and humans. Beginning with the comparison of the three non-human species, we observed prominent clustering of transcripts between the normal brain and tumors from each species. Notable glioma-associated genes were up- and down-regulated uniquely in each species, suggesting specific influences of the tumor host on glioma biology. The rat tumors showed the lowest percentage of unique DEGs and the greatest percent of genes overlapping in all three species within the total rat DEG set. Conversely, the canine tumors showed the highest percentage of unique DEGs and the lowest percent overlap. Upon inclusion of the human glioma data characterized by up-regulation of the PDGF/PDGFR signaling axis, a similar pattern continued with the rat tumors having the smallest percentage of unique DEGs, and the largest percent overlap of the common DEGs within the total rat DEG set. The human and canine tumors showed the greatest percentage of unique DEGs and lowest percent overlap of the common DEGs, perhaps representing greater molecular heterogeneity in these tumors. Inter-species comparisons between non-human and human tumors revealed over 80% overlap in the total increased and decreased DEGs in each case. Using a subset of glioma-related genes, function annotation analysis generated graded enrichment scores for specific biological pathways and revealed the greatest core alterations in the signal transduction and migration/invasion pathways. The glioma-associated gene set was compared next by fold change in expression across the four species. This revealed a remarkably similar overall pattern of increased and decreased expression with a few notable exceptions in genes known to mediate key elements of glioma biology; specifically, angiogenesis, immune function, invasion, and self-renewal. GSEA incorporating cell type signatures for brain and infiltrating cell populations, revealed evidence of mixed immune cell, astrocytic, and oligodendrocytic contributions across the four species. Taken together, these results present a cross-mammalian set of neoplastic alterations in PDGFRA-overexpressing glioma, as well as unique contributions and possible limitations related to the tumor host species.

The findings of this study represent an initial step towards understanding the core elements that define what it means to be a glioma, independent of the tumor host. In addition, the interplay between specific aspects of glioma biology (e.g. PDGFRA overexpression) and tumor host features is revealed across four different mammalian species. PDGFRA is known to be a critical gene in gliomagenesis and progression. Numerous studies have identified autocrine/paracrine loops present in human and murine tumors, corroborating the importance of this signaling pathway in gliomas^19–21^. While a mechanistic explanation of how these signaling changes drive specific and core elements of glioma biology is beyond the scope of this study, there are numerous intriguing findings from this study that warrant discussion.

This study is also the first step in characterizing TV-A rat gliomas, and their comparison with human tumors. In the PDGF-driven TV-A rat tumors, the glioma-related genes IGFBP2 and BMP7 were uniquely under-expressed compared to the other three species. The protein products of these transcripts have been shown to be important mediators of tumor cell growth and self-renewal^40,41^. While multiple other genes were uniquely up- and down-regulated in the rat tumors (e.g. RET, SOX9), overall, the rat gliomas showed the smallest percentage of unique DEGs (only 7% of up-regulated DEGs, 9% of down-regulated DEGs), and the largest percent overlap with the common DEGs (32% of up-regulated DEGs, 34% of down-regulated DEGs). This suggests that the rat tumors have a high degree of biological overlap with the core glioma biological patterns. To date, only one other transgenic rat brain tumor model has been described where tumors form i*n situ* within the brain^42,43^. In this model, the S-100β promoter was used to drive v-erbB expression, and these rats were found to develop spontaneous astrocytic and oligodendroglial brain tumors with varying penetrance and histologic grades and patterns. Transcriptional analyses of these tumors were not available to compare to our results. Additionally, the v-erbB gene alters EGF-related signaling likely differentiating these tumors from those in our study, which featured PDGF/PDGFR signaling. Regarding broader differences in the anatomy and physiology of the rat brain, numerous studies have analyzed and compared structural, behavioral, and immunologic findings in rats compared to mice and humans in the context of neoplastic, infectious, traumatic, and other disease conditions^6,15,42,44–49^. Many of these studies suggest that the rat brain and immunological environments more closely mimic humans compared to mice. Notably, the GSEA revealed that only the rat tumors shared enrichment of the M2 macrophage signature with the human tumors. Recent studies reveal a crucial role for glioma-infiltrating macrophages and microglia in glioma biology, and emerging evidence suggests M2-like cell features are associated with tumor-supporting functions^37,50–53^. Our ongoing and prior^15^ studies using this TV-A rat model system are confirming the close resemblance of rat and human gliomas. While the relative degree of intra-tumor heterogeneity remains an important consideration when comparing model systems to humans, a broader analysis of the transcriptional landscape found in multiple genetically-tailored tumor types in this rat model has yet to be performed.

Gliomas initiated in TV-A transgenic mice have been studied extensively^25,51,54–59^. In particular, the gene expression patterns in proneural-like and mesenchymal-like tumors have been compared and analyzed to correlate with observations made in human tumors in an effort to understand sequential driver events in gliomagenesis^9^. Multiple prior and ongoing studies have used this versatile murine model system for pre-clinical development and testing of new therapies, many of which have progressed into clinical trials^51,60,61^. In this study, the glioma-related genes POSTN and CSFR1 were found to be uniquely under-expressed compared to the other three species. The protein products of these transcripts have been shown to be important mediators of glioma-infiltrating macrophage and microglia (GIM) infltration and function in human tumors^36,51,57,62^. This finding may have implications for pre-clinical testing of treatments aimed at GIMs using this tumor model. The GSEA reinforces this consideration as the mouse tumors did not show enrichment of the M2 macrophage signature. Other genes uniquely altered in mouse gliomas (e.g. NOTCH4, FGF10, JUNB, RHOB) may contribute further to murine-specific differences in glioma biology. Notable studies exploring unique aspects of other diseases in mice have reported important differences with humans^63,64^.

Much less in known about the transcriptional variations present in canine gliomas. To date, there have been relatively few genomic or transcriptomic analyses of canine brain tumors^17^. In this study, some important glioma-related genes were not differentially expressed in PDGFRA-overexpressing canine gliomas, including HIF1-α, CTLA4, DLL3, and TNFRSF12A, important mediators of angiogenesis, immune evasion, self-renewal, and invasion^65–68^ in the tumor microenvironment. Notably, CTLA-4 is found only on T-cells; therefore, low levels of this transcript may represent differences in the T-cell populations within canine gliomas compared to other species. Significant variability in the infiltrating cell population signatures was demonstrated by the GSEA, suggesting important differences in the mammalian host inflammatory responses to glial tumors. Current theory suggests that canine gliomas, which are the only other mammalian species known to develop spontaneous gliomas, are the closest biological cousin to human tumors. Interestingly, dogs develop brain tumors at a rate of 3–5 times that observed in humans, despite their significantly shorter life spans^69^. Major efforts are ongoing to examine the applicability of canine gliomas for human brain tumor research, as well as investigate approaches for parallel development of new treatment strategies.

Human glioma is a markedly heterogeneous and treatment resistant disease, which relies on components of the host central nervous and immune systems to develop and evolve^70–72^. As such, tumors that develop *in situ* within the milieu of the host brain are more likely to accurately represent the human disease, despite potential differences in overall tumor heterogeneity^7^. Characterizing and recreating core neoplastic alterations within the tumor-host ecosystem therefore represents an important step in establishing accurate glioma models. This study compared *in situ*-formed tumors in rats, mice, dogs, and humans with a shared biological feature (PDGFRA overexpression), increasing the relevance of the cross-species comparisons and relating these finding to human glioma; more specifically, the proneural molecular subtype^73^. The findings from this study suggest that the gliomas studied from all three non-human species closely resemble human tumors, as suggested by the ~80% overlap in DEGs in each case and similar pattern of glioma-associated DEGs. Yet, the de-convolved data analysis based on cell-type gene expression signatures revealed potentially important differences supported by subtle variability in infiltrating cell populations and specific transcripts related to immune function (e.g. POSTN^37^).

Equally important to accurate glioma models is their ability to advance our understanding of glioma biology. Some intriguing aspects of the core gene set identified in this study include the well-studied glioma-related genes ANGPT2, CD44, SPP1, STAT3, PDGRFA, and TOP2A (all up-regulated), and BRSK1, DKK3, FGFR2, MAPK9, MEF2C, and PTEN (all down-regulated). While this is only a small subset of the 563 overlapping DEGs in the four species and the relative timing of each gene alteration is not known, this set of up- and down-regulated genes provides new insights into the complex of altered elements that promote and maintain high-grade gliomas. Further insights may be gleaned from mapping this gene set to chromosomal locations and/or specific mutations present in these tumors. The pathway enrichment analysis performed here is another method of relating the observed gene expression patterns to the biology driving these tumors. This analysis revealed the significant contributions of signal transduction and migration/invasion pathways. Prior studies using systems biology approaches have found similar results related to gliomas in humans and mice^74–77^, but this is the first description of enriched biological pathways across four mammalian glioma species.

The full complexity and biological mechanisms underlying the formation and evolution of human gliomas remains incompletely understood. In addition, the lack of experimental systems that accurately and completely represent the human disease remains a major obstacle to predictive testing of new treatments for this disease. As this information becomes better known, it will be equally important to develop an expanded understanding of the relative value and limitations of corresponding experimental models. Accordingly, the current study sought to uncover new information regarding established and emerging glioma models, and how these relate to our current understanding of the human disease.

There are multiple possible future directions as well as some limitations of this work. Potential next steps include comparative analysis of genomes, transcriptomes and proteomes, expanding the analyses to cover other tumor subtypes and cell type-specific analyses, and comparative treatment studies exploring cross-species variation in responses and adverse events. A limitation of the current study relates to the different gene expression analysis platforms utilized (microarray vs. RNA sequencing), which limited the total number of transcripts included and the range of fold change assessed. The core glioma components identified in this study likely only represent a subset of the ‘true core components’ due to these limitations as well as those related to tumor sampling biases and the selected tumor types. Our analysis therefore could not examine the comprehensive transcriptome of these tumors, and may have excluded important differences and similarities. We also chose to select a subset of glioma-associated genes based on a broad review of the literature to include in the pathway analysis and cross-species comparison of DEG fold changes. This was done to facilitate and contextualize the analysis of the overlapping and species-specific findings. One could imagine other approaches to this analysis with unrestrained transcriptional analyses or by selecting other DEG sets such as those related to immunologic signaling or DNA repair mechanisms exclusively. Despite these study characteristics, valuable insights regarding conserved glioma biology can be derived from these large DEG data sets, and the results provide valuable information regarding the comparative value and applicability of these mammalian glioma models.

## Electronic supplementary material

Supplementary Information

Dataset 1

Dataset 2

Dataset 3

Dataset 4

Dataset 5

Dataset 6

Dataset 7
