# Supplementary material for: Cross-species transcriptional analysis reveals conserved and host-specific neoplastic processes in mammalian glioma
Source: Sci Rep. 2018 Jan 19;8:1180. doi: 10.1038/s41598-018-19451-6 (PMC5775420; doi:10.1038/s41598-018-19451-6)
Supplement: Supplementary file 1 — Supplementary Information [file 41598_2018_19451_MOESM1_ESM.pdf]

Cross-species transcriptional analysis reveals conserved and host-specific neoplastic processes in mammalian glioma

Nina P. Connolly[1*], Amol C. Shetty[2*], Jesse A. Stokum[1], Ina Hoeschele[3], Marni B. Siegel[4], C. Ryan Miller[4], Anthony J. Kim[1,6], Cheng-Ying Ho[5,6], Eduardo Davila[6,7], J. Marc Simard[1], Scott E. Devine[2,6,8], John H. Rossmeisl[9,10], Eric C. Holland[11], Jeffrey A. Winkles[6,12,13] & Graeme F. Woodworth[1,6]

1 Department of Neurosurgery, University of Maryland School of Medicine, Baltimore, Maryland
2 Institute for Genome Sciences, University of Maryland School of Medicine, Baltimore, Maryland
3 Virginia Bioinformatics Institute and Department of Statistics, Virginia Tech, Blacksburg, Virginia
4 Departments of Pathology and Laboratory Medicine, Neurology, and Pharmacology, Lineberger Comprehensive Cancer Center and Neuroscience Center, University of North Carolina, Chapel Hill, North Carolina
5 Department of Pathology, University of Maryland School of Medicine, Baltimore, Maryland
6 Marlene and Stewart Greenebaum Comprehensive Cancer Center, University of Maryland School of Medicine, Baltimore, Maryland
7 Department of Microbiology and Immunology, University of Maryland School of Medicine, Baltimore, Maryland
8 Department of Medicine, University of Maryland School of Medicine, Baltimore, Maryland
9 Department of Small Animal Clinical Sciences, Virginia-Maryland College of Veterinary Medicine, Blacksburg, Virginia
10 Wake Forest University Baptist Health Comprehensive Cancer Center, Brain Tumor Center of Excellence, Winston-Salem, North Carolina
11 Fred Hutchinson Cancer Research Center, University of Washington, Seattle, Washington
12 Department of Surgery, University of Maryland School of Medicine, Baltimore, Maryland
13 Center for Vascular and Inflammatory Diseases, University of Maryland School of Medicine, Baltimore, Maryland

* Co-first authors

Corresponding Author:
Graeme F. Woodworth, M.D., F.A.C.S.
Departments of Neurosurgery, Diagnostic Radiology, Anatomy & Neurobiology
Greenebaum Comprehensive Cancer Center
University of Maryland School of Medicine
22 S. Greene St., S-12-D
Baltimore, MD 21201
gwoodworth@som.umaryland.edu
Supplementary files include two tif figures that give supporting evidence for controls.

Additionally, several tables containing the gene names and results from microarray analysis.

File formats submitted:

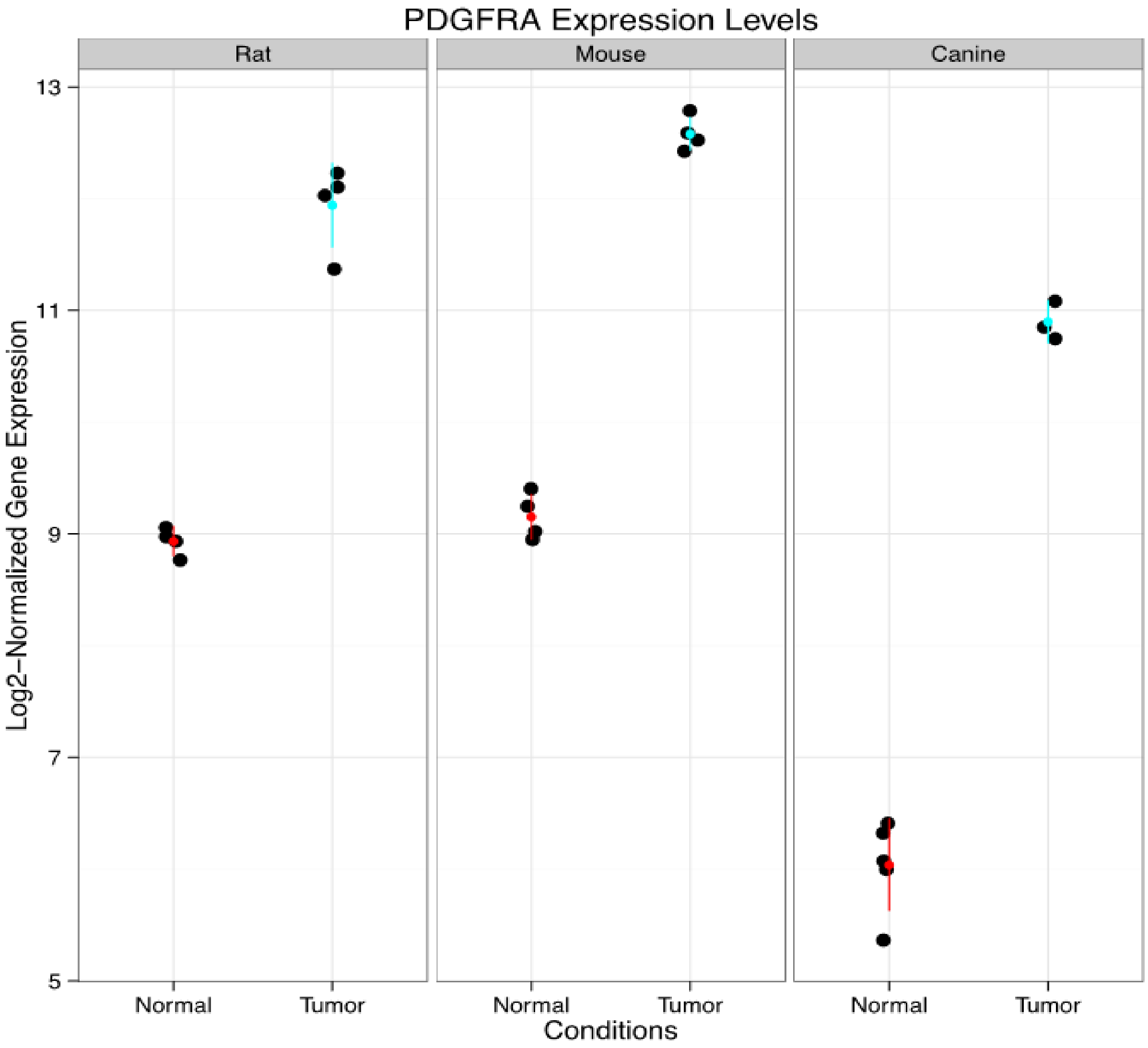

Supplementary Figure 1. Control normal brain and tumor tissue PDGFR-α gene expression levels in rat, mouse, and canine gliomas. Each filled black circle represents an individual sample, and the red and blue dots and lines represent the mean +/- standard deviation.

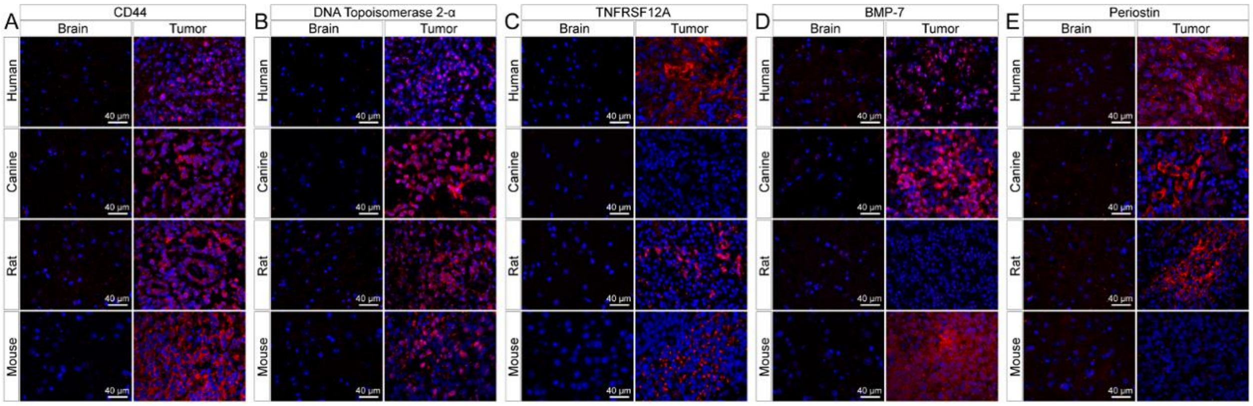

Supplementary Figure 2. Analysis of CD44, DNA TOP2-α, TNFRSF12A, BMP-7, and POSTN (periostin) protein expression in normal brain and brain tumor tissue by immunofluorescence microscopy. Immuno-labeling of **(A)** CD44 and **(B)** DNA TOP2-α protein in rat, mouse, canine, and human normal brain and glioma tissues. These two genes were commonly overexpressed in glioma tissue compared to normal brain tissue. Immuno-labeling of **(C)** TNFRSF12A (Fn14), **(D)** BMP-7, and **(E)** Periostin in rat, mouse, canine, and human brain and glioma tissues. These three genes were variably expressed in the four species with low TNFRSF12A expression in canine tumors, low BMP7 expression in rat tumors, and low POSTN expression in mouse tumor tissue.

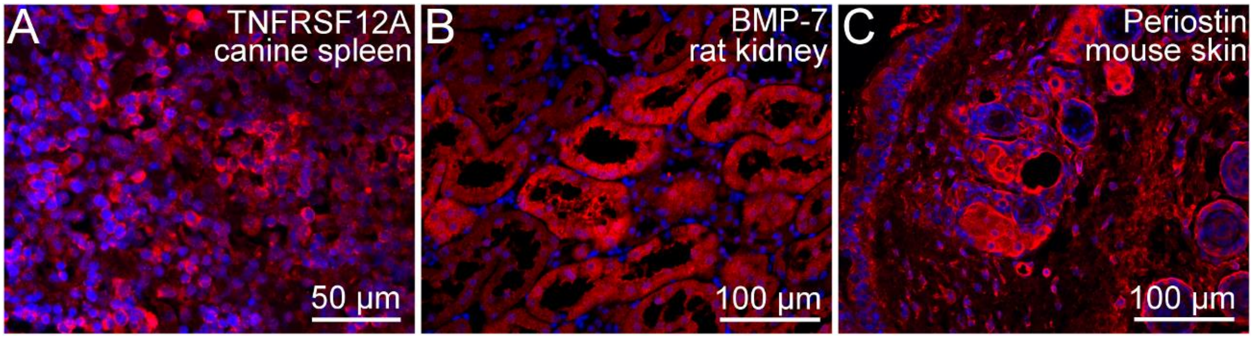

Supplementary Figure 3. Antibody species cross-reactivity validation by immunofluorescence microscopy. Immunolabeling of TNFRSF12A, BMP-7, and POSTN in canine spleen, rat kidney, and mouse skin, respectively, confirms that the antibodies used for the data in Supplementary Figure 2 can cross-react with the proteins expressed by these species.

Excel Spreadsheets:
Supplementary Table 1: Differentially expressed genes identified for the Rat Microarray
Supplementary Table 2: Differentially expressed genes identified for the Mouse Microarray
Supplementary Table 3: Differentially expressed genes identified for the Canine Microarray

Supplementary Table 4: Differentially expressed genes identified for the Human Microarray
Supplementary Table 5: Overlap of differentially expressed genes across all four species

- **Sheet 1:** Up-regulated genes
- **Sheet 2:** Down-regulated genes

Supplementary Table 6: 125 up-/down-regulated genes selected based on prior knowledge of GBM specific genes
Supplementary Table 7: Pathway clusters and gene assignment for GBM specific genes

- **Sheet 1:** Pathway clusters based on Gene Ontology enrichment of 125 GBM specific genes
- **Sheet 2:** Assignment of GBM specific genes to Pathway Super Groups for figure 7
